# Higher Abdominal Adiposity Is Associated With Lower Muscle Strength in Chilean Adults

**DOI:** 10.3389/fnut.2022.812928

**Published:** 2022-02-23

**Authors:** Ana Cristina Palacio, Ximena Díaz-Torrente, Daiana Quintiliano-Scarpelli

**Affiliations:** Carrera de Nutrición y Dietética, Facultad de Medicina– Clínica Alemana, Universidad del Desarrollo, Santiago, Chile

**Keywords:** muscle strength, relative handgrip strength, overweight, abdominal obesity, dynamometry, adulthood

## Abstract

Handgrip strength (HGS) is a well-established indicator of muscle strength and a reasonable clinical predictor of metabolic health and diseases. This study explores the association between relative muscular strength and abdominal obesity (AO) in healthy Chilean adults. A convenience sample was recruited (*n* = 976) between 2018 and 2020. The HGS was determined by dynamometry. The anthropometry (weight, height, waist, and mid-arm circumference) and physical activity were also measured. The relative HGS (RHGS) was calculated by dividing the maximum HGS of the dominant hand by the body mass index. The AO was defined as a waist circumference (WC) >88 cm for women, and >102 cm for men. From the sample, 52.6% were women, 56.4% had excessive weight, and 42.7% had AO. The absolute and RHGS were greater in men compared to women (*p* < 0.001) and were decreased with age in both sexes. We observed a moderate negative correlation between WC and RHGS (*rho* = −0.54, and *rho* = −0.53, for men and women, respectively). The RHGS was lower in individuals with AO, independent of age and sex (*p* < 0.05). For each cm increase in WC, the odds of low RHGS (<25th percentile) increased by 12 and 9% for men and women, respectively. The AO is related to higher odds for low RHGS (OR: 1.72; 95% CI: 1.23–2.41). In our sample of healthy adults, a higher AO was associated with a lower muscle strength measured by dynamometry.

## Introduction

The relationship between adipose tissue and muscle function has attracted interest in recent years (1–9). Increasing the central adipose tissue (i.e., abdominal obesity) may reduce muscle function through a complex interplay of factors, such as enhanced levels of inflammatory mediators and insulin resistance (10). There is some evidence that low handgrip strength (HGS), a proxy for general muscle strength, is associated with age-related unhealthful weight gain, obesity-related comorbidities, and metabolic complications (11–14).

The relationship between muscle strength and adiposity has been explored, but the strength of this association varies according to the assessed anthropometric measure. Findings from 8,441 participants from the European Prospective Investigation into Cancer-Norfolk Study showed that higher body mass index (BMI) was associated with stronger HGS, but a larger waist circumference (WC) was associated with a weaker HGS (1). Recent evidence has shown that abdominal fat contributes to a greater loss of muscle strength *via* neuroendocrine dysregulations (2, 3). In addition, there are sex differences related to abdominal fat deposition patterns. Women accumulate more subcutaneous fat, compared to men who accumulate more visceral fat. Such differences could also play a role in the sex-specific rate of muscle strength decline (4).

The use of the absolute HGS might introduce bias as compared with the relative handgrip strength (RHGS) defined as absolute HGS divided by BMI (HGS/BMI). When muscle strength is compared without correcting for body mass, women with obesity present a higher level of absolute muscle strength (5, 6) compared with overweight and normal weight women. Furthermore, when adjusted for BMI, women with obesity present lower RHGS (7). Thus, RHGS might be an interesting and convenient tool to use in a clinical practice to classify the subjects with reduced physical function (8) and to determine the risk factors in cardiometabolic disease (9).

In recent population-based studies, stronger correlations have been observed between RHGS and cardiovascular biomarkers, compared to absolute HGS and dominant HGS (6). The RHGS, which not only reflects the maximal HGS of each hand but also minimizes the confounding effect of body size, appears to be the best marker of cardiometabolic risk among various HGS indices evaluated to date (10). Considering this framework, the aim of this study was to explore the association between RHGS and AO in healthy Chilean adults. Additionally, we calculated the cut-off point values for RHGS according to age and sex.

## Materials and Methods

### Design and Sample

An observational, cross-sectional study was conducted in a non-random sample of healthy Chilean adult subjects (18–64 years old) from the Metropolitan and Valparaiso regions of Chile. Individuals were recruited between June 2018 and November 2020 in shopping centers, the waiting rooms of health centers, supermarkets, banks, universities, gyms, and private companies. The exclusion criteria included illness, hospitalization within the past month, subjects with any pathology that affected strength, and sensitivity of upper extremities, history of upper limb injury or deformity with motor impairment, neurological disorders involving the hand (e.g., stroke, Parkinson's disease, and neurodegenerative disorders), and others who routinely perform a high-demand upper-extremity work, professional sportsmen/women, and being a foreigner.

The study protocol followed the Helsinki Declaration and was approved by the Ethics Committee of the Universidad del Desarrollo – Facultad de Medicina Clínica Alemana. A total of 1,016 subjects consented to participate in the study; of these 1,004 completed the handgrip assessments and 988 had the WC measured. The final sample was composed of 976 participants.

### Instruments and Data Collection

#### Assessment of Muscle Strength

The Jamar® digital hand dynamometer (Patterson Medical, Warrenville, IL, USA) was used to measure HGS. The participants were seated on a standard height chair without armrests and positioned per the American Society of Hand Therapists' recommendation (11). The subjects were seated with one shoulder adducted and neutrally rotated, elbow flexed at 90°, the forearm in a neutral position, and the wrist in 0°-30° dorsiflexion and 0°-15° ulnar deviation. The grip handle of the dynamometer was adjusted based on participant hand size to obtain optimal grip position. The test was performed with standardized verbal instructions (i.e., “*one, two, three, squeeze… harder… harder…*”), and the participants were asked to use the non-dominant hand first, followed by the dominant hand. Three measurements of HGS were taken for each hand, with 10–20 s of rest between measurements to avoid fatigue. Absolute HGS was considered as the maximum strength of the dominant hand achieved in any of the three attempts. The RHGS was defined as the absolute HGS divided by BMI and was expressed as kg/kg/m^2^. We defined low relative muscle strength values as under the 25th percentile by sex.

#### Anthropometric Measures

Anthropometric measures (weight, height, waist, and mid arm circumferences) were conducted by trained evaluators following the National Health and Nutrition Examination Survey protocol (12). The participants were weighed using a SECA 8031 scale (Hamburg, Germany) with 100-g precision in light clothing and bare feet. Height was measured in the Frankfurt position using a SECA 2131 stadiometer (Hamburg, Germany) with 1-mm precision. The BMI was calculated in kg/m^2^ using the measured weight and height. The participants were categorized as normal weight when the BMI was <25 kg/m^2^, overweight with a BMI between 25 and 29.9 kg/m^2^, and obese with a BMI ≥ 30 kg/m^2^.

The WC was determined using a flexible metric tape at the midpoint between the iliac crest and the last rib. The participants remained standing with arms alongside the body and the trunk that is free of clothing. The measurement was performed with the relaxed abdomen at the end of expiration. The AO was defined as a WC >88 cm for women and >102 cm for men (13). The AO was analyzed by two variables, in a quantitative procedure by WC in cm and in categoric variable by AO classification.

#### Physical Activity

Type of activity, frequency, and duration were self-reported through a questionnaire developed for this research based on the International Physical Activity Questionnaire (IPAQ) (15). The type of the physical activity was classified by the research group according to the criteria of the World Health Organization (WHO) (14). The subjects who performed <150 min per week of moderate intensity aerobic physical activity and those who reported not engaging in regular physical activity at the examination moment were classified as sedentary. Those who performed 150 min or more of moderate physical activity or 75 min a week of vigorous physical activity were classified as active.

### Statistical Analysis

Descriptive data from categorical variables were expressed in relative and absolute frequencies. Numeric data were described as medians, interquartile ranges, and percentiles (p25, p50, p75, p90, p95), according to sex and age. The Shapiro–Wilk test was used to determine the normality of the distribution. For bivariate analysis assessing sex differences in covariates, the chi-square, Student *T*-test, Kruskal–Wallis, and Mann–Whitney *U* tests were used depending on variable type and distribution. To correlate WC (cm) and RHGS (kg/kg/m^2^), the Spearman's rank correlation coefficient was used. The multivariate logistic regression was used to assess the association between AO and low RHGS, stratified by sex. Models were adjusted for the following covariates: age (years), physical activity status, and mid-arm circumference (cm) regardless of their levels of significance. This is based on the available evidence that the aforementioned variables have been associated with the outcomes of our study (10, 16, 17). Goodness of fit was measured with the Hosmer–Lemeshow test. Alpha was set at *p* < 0.05. Statistical analysis was carried out using STATA 16.1 for Mac (StataCorp LLC, College Station, TX, USA).

## Results

Table 1 provides descriptive statistics of the sample, overall, and by sex. Almost 50% of the sample was under 30 years of age. Regarding the nutritional status, 55.4% were overweight and 42.7% presented an AO. Nearly a third of the participants (34.0%) were sedentary. For all these variables, there was a significant difference by sex (*p* < 0.001). In relation to the dominant hand, 92.7% were right-handed, with no difference by sex (*p* = 0.413). Men presented higher HGS (around 20 kg of difference) than women in both hands, and right hands had higher values in both sexes (*p* < 0.001).

**Table 1:** General characteristics of the sample.

**Variables**	**Overall (*n* = 976)**	**Men (*n* = 463)**	**Women (*n* = 513)**
Age group, *n* (%)			
<30 y	485 (49.7)	258 (55.7)	227 (44.3)
30–44 y	241 (24.7)	125 (27.0)	116 (22.6)
≥45 y	250 (25.6)	80 (17.3)	170 (33.1)
Nutritional status, *n* (%)			
Normal weight	435 (44.6)	156 (33.7)	279 (54.4)
Overweight	369 (37.8)	210 (45.3)	159 (31.0)
Obesity	172 (17.6)	97 (21.0)	75 (14.6)
Mid arm circumference (cm)^a^	31.0 (28.3–34.0)	32.6 (30.5–35)	29.5 (27.0–32.0)
WC (cm)^a^	87.8 (78.1–97.0)	92.0 (84.0–100.5)	82.0 (75.0–93.4)
Abdominal obesity, *n* (%)	417 (42.7)	170 (36.7)	247 (48.2)
HGS			
Right hand (kg)^a^	35.2 (28.1–47.7)	48.0 (42.4–54.5)	28.4 (25.1–31.8)
Left hand (kg)^a^	32.8 (26.3–44.8)	45.5 (39.6–50.7)	26.7 (23.4–30.4)
RHGS (kg/kg/m^2^)^a^	1.41 (1.1–1.80)	1.80 (1.52–2.12)	1.14 (0.98–1.32)
Physical activity, *n* (%)			
Sedentary	332 (34.0)	118 (25.5)	214 (41.7)
Active	644 (66.0)	345 (74.5)	299 (58.3)

*BMI, body mass index; WC, waist circumference; HGS, handgrip strength; RHGS, relative handgrip strength*.

a *Variables are described as median and percentiles 25th and 75th*.

*For all the variables presented, there was a statistically significant difference by sex (p < 0.001)*.

Table 2 shows percentile values of RHGS by age and sex. In general, values of RHGS were lower in women and decreased with age, in both sexes.

**Table 2:** Percentile values of relative handgrip strength (kg/kg/m^2^) by sex and age.

**Percentiles**	**25**	**50**	**75**	**90**	**95**
Men					
Age group					
<30 y	1.7	2.0	2.3	2.5	2.7
30–44 y	1.5	1.7	2.0	2.3	2.4
≥45 y	1.4	1.5	1.8	1.9	2.0
Women					
Age group					
<30 y	1.1	1.2	1.4	1.6	1.7
30–44 y	0.9	1.1	1.3	1.5	1.7
≥45 y	0.9	1.0	1.2	1.3	1.4

**Figure 1:**
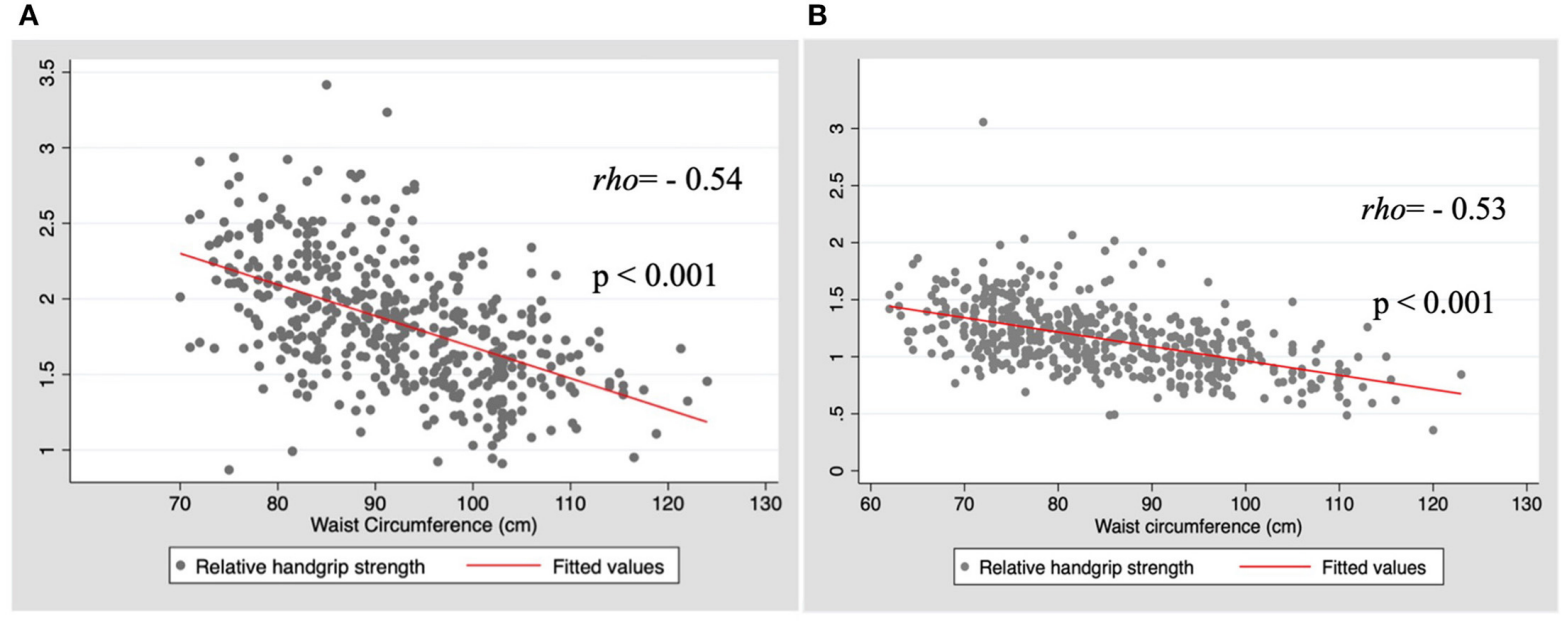
Correlation between relative handgrip strength (HGS) and waist circumference by sex. **(A)** Men; **(B)** Women.

The results of the correlational analysis between HGS and WC are shown in Figure 1. First, we conducted an analysis of maximum HGS of the dominant hand and WC; a weak/null correlation was observed *(rho* = 0.03, *p* = 0.554; and *rho* = 0.05, *p* = 0.280, for men and women, respectively). When considered the RHGS, in both sexes, the correlation was moderate, negative, and significant (*rho* = −0.54, *p* < 0.001; and *rho* = −0.53, *p* < 0.001, for men and women, respectively).

Table 3 presents the RHGS values by AO classification according to sex and age. The subjects with AO had significantly lower RHGS in all age groups in both sexes (*p* < 0.05).

**Table 3:** Relative handgrip strength (kg/kg/m^2^) by abdominal obesity status, according to sex and age.

	**Without abdominal obesity**	**With abdominal obesity**	***p*-value**
Men			
Age group			
<30 y	2.0 [1.7–2.3]	1.6 [1.4–1.7]	**<0.001**
30–44 y	1.8 [1.6–2.1]	1.5 [1.4–1.7]	**<0.001**
≥45 y	1.7 [1.4–1.8]	1.4 [1.1–1.5]	**<0.001**
Women			
Age group			
<30 y	1.3 [1.1–1.4]	1.0 [0.8–1.2]	**<0.001**
30–44 y	1.2 [1.0–1.4]	1.0 [0.8–1.2]	**<0.001**
≥45 y	1.2 [1.0–1.3]	0.9 [0.8–1.1]	**<0.001**

*Variables are described as median and percentiles 25th and 75th. Bold indicates statistically significant differences*.

The association between low RGHS (<25th percentile) and AO was significant both in unadjusted and adjusted models. It can be seen from the data in Table 4 that, for each cm increase in WC, the odds of presenting low RHGS were increased by 12 and 9% for men and women, respectively (both *p* < 0.001). In addition, having AO increased the probability of presenting a low RHGS in the overall sample by 72%. This association remained significant for women only in adjusted models (OR: 1.95; 95% CI: 1.16–3.27).

**Table 4:** Associations between low relative handgrip strength and abdominal adiposity.

**Low RHGS^**a**^ (kg/kg/m^**2**^)**	**Unadjusted**			Adjusted^*^		
	**OR**	**95% CI**	***p*-value**	**OR**	**95% CI**	***p*-value**
Overall						
WC (cm)	1.10	1.08–1.11	**<0.001**	1.09	1.07–1.12	**<0.001**
Abdominal obesity	2.98	2.21–4.02	**<0.001**	1.72	1.23–2.41	**0.002**
Men						
WC (cm)	1.11	1.09–1.14	**<0.001**	1.12	1.08–1.16	**<0.001**
Abdominal obesity	2.20	1.43–3.38	**<0.001**	1.57	0.97–2.52	0.065
Women						
WC (cm)	1.10	1.08–1.13	**<0.001**	1.09	1.06–1.12	**<0.001**
Abdominal obesity	4.19	2.70–6.52	**<0.001**	1.95	1.16–3.27	**0.012**

*WC, waist circumference; CI, confidence interval*.

a *p25th cutoff point: 1.52 kg/kg/m^2^ for men, and 0.98 kg/kg/m^2^ for women*.

* *Adjusted by age (years), physical activity status, and mid-arm circumference (cm)*.

*Bold indicates statistically significant differences*.

## Discussion

The present study explored the associations of AO and muscle strength. We hypothesized and found that the participants with AO would present lower muscle strength. As expected, compared to the women, the men presented higher HGS, and HGS decreased with age in both sexes. Another study carried out in Chile (16) and studies from other countries showed similar results (17, 18). An additional objective proposed for this investigation was to determine the RHGS cut-off points according to age and sex. These cut-off points could be used to evaluate the risk of sarcopenia at ages, which is uncommon to find in this condition. More studies are needed to confirm the proposed percentiles points.

Several studies have shown that aging is associated with a decline in HGS, and these studies highlighted the fact that an increase in fat mass contributes to deterioration of HGS in older adults (19, 20). These processes can be viewed as a cascade of events, beginning with aging, which is associated with a greater muscle fat infiltration (21, 22). Beyond its corresponding effect on health, excess adiposity has a harmful impact on muscle quality and quantity (19); the convergence of aging and fat mass may create a perfect storm for skeletal muscle catabolism (23).

In this study, the subjects with AO had lower HGS than the subjects without AO, in both sexes. This result is in accordance with other studies (1–3, 19). In particular, Keevil et al. showed that, for every 10-cm increase in WC, muscle strength will decrease by 3.56 kg in middle-aged and older men (1). In our study, we found that AO was associated with a low RHGS, which was more evident in men than in women. However, the association was no longer significant in men when the model was further adjusted for physical activity and mid-arm circumference. This could be explained by the effect of the exercise among those with greater muscle mass, as is the case of the men in our study (24). In our study, when maximum HGS and WC were analyzed, a weak/null correlation was detected; however, when adjusted by BMI, we observed a moderate, negative, and significant association between WC and RHGS in both sexes. We also found that, for both sexes, as WC increased, RHGS decreased. Accordingly, it is recommended to maintain a healthy WC. A possible explanation for this phenomenon is that excessive adiposity can downregulate the anabolic actions of testosterone (25), growth hormones (26), and insulin (27), which can contribute to a progressive loss of muscle mass and its functionality in both sexes. Additionally, excessive adipose tissue can induce a pro-inflammatory state by the action of several cytokines (e.g., higher plasma concentrations of tumor necrosis factor-alpha and interleukin-6), which is associated with lower muscle strength (28).

Furthermore, Otten et al. indicate that the grip strength cannot be considered an indicator of whole-body strength in obese individuals because the relationship between obesity and upper and lower extremity strength differs (29). Since absolute HGS is closely related to body mass, using the absolute handgrip strength as an indicator of muscle strength without correction for body mass may explain these conflicting results (30, 31). Thus, RHGS may represent the muscle strength adjusted for body size, which provides more accurate information for sarcopenic obesity screening (32).

Our results should be interpreted with the following limitations. First, we did not measure body composition, which is a more specific indicator of adiposity (33). Nevertheless, WC is an adequate measure for the evaluation of adiposity in adult subjects (34), because body composition in this population can change independently of variations in total mass (2). Second, the results cannot be extrapolated for the entire Chilean adult population since the sample consisted only of urban subjects residing in two regions of Chile, and our sample presented a higher physical activity level than the national statistics indicate (35). On the other hand, the strengths of our study include the use of a relatively simple and non-invasive methodology for muscle strength measurement that is assessed with a standardized protocol. In addition, considering the well-established sex differences in strength, we stratified all analyses by sex. Our findings mainly support an association of data, and future longitudinal design studies should explore the cause-effect relationship between AO and low RHGS. Finally, this study offers the first approximation to reference values for RHGS in adults, which is novel and important for Chile and for future comparison studies.

## Conclusion

In conclusion, our findings contribute to the understanding of the association of AO and low RHGS in the adult population. The WC measurement seems to be a good parameter to identify a low RHGS, since both studied measures revealed associations with lower muscle strength. Additionally, the cut-off points for RHGS by sex and age may be a clinically useful tool to contribute to the evaluation of risk of sarcopenia in young and middle-aged adults. In view of these findings, the WC and HGS measurements should be standard protocols for healthcare professionals to carry out the prevention and adequate treatments, starting in adulthood.

## Data Availability Statement

The raw data supporting the conclusions of this article will be made available by the authors, without undue reservation.

## Ethics Statement

The studies involving human participants were reviewed and approved by Ethics Committee of the Universidad del Desarrollo – Facultad de Medicina Clínica Alemana (record number 2018/42), dated Jun 13, 2018. The patients/participants provided their written informed consent to participate in this study.

## Author Contributions

ACP, XD-T, and DQ-S: conceptualization, investigation, methodology, validation, writing—original draft, and writing—review and editing. ACP: supervision. DQ-S: analysis and software. All authors contributed to the article and approved the submitted version.

## Conflict of Interest

The authors declare that the research was conducted in the absence of any commercial or financial relationships that could be construed as a potential conflict of interest.

## Publisher's Note

All claims expressed in this article are solely those of the authors and do not necessarily represent those of their affiliated organizations, or those of the publisher, the editors and the reviewers. Any product that may be evaluated in this article, or claim that may be made by its manufacturer, is not guaranteed or endorsed by the publisher.
